# Optimized bacterial community characterization through full-length 16S rRNA gene sequencing utilizing MinION nanopore technology

**DOI:** 10.1186/s12866-024-03208-5

**Published:** 2024-02-16

**Authors:** Alessandro Bertolo, Ezra Valido, Jivko Stoyanov

**Affiliations:** 1 SCI Population Biobanking & Translational Research Group, Swiss Paraplegic Research, Nottwil, Switzerland; 2 Department of Orthopaedic Surgery, University of Bern, Bern Inselspital, Bern, Switzerland; 3 Institute of Social and Preventive Medicine, University of Bern, Bern, Switzerland

**Keywords:** Bacterial DNA, 16S rRNA gene-based sequencing, V1–V9 region, Nanopore sequencing

## Abstract

**Background:**

Accurate identification of bacterial communities is crucial for research applications, diagnostics, and clinical interventions. Although 16S ribosomal RNA (rRNA) gene sequencing is a widely employed technique for bacterial taxonomic classification, it often results in misclassified or unclassified bacterial taxa. This study sought to refine the full-length 16S rRNA gene sequencing protocol using the MinION sequencer, focusing on the V1–V9 regions. Our methodological enquiry examined several factors, including the number of PCR amplification cycles, choice of primers and Taq polymerase, and specific sequence databases and workflows employed. We used a microbial standard comprising eight bacterial strains (five gram-positive and three gram-negative) in known proportions as a validation control.

**Results:**

Based on the MinION protocol, we employed the microbial standard as the DNA template for the 16S rRNA gene amplicon sequencing procedure. Our analysis showed that an elevated number of PCR amplification cycles introduced PCR bias, and the selection of Taq polymerase and primer sets significantly affected the subsequent analysis. Bacterial identification at genus level demonstrated Pearson correlation coefficients ranging from 0.73 to 0.79 when assessed using BugSeq, Kraken-Silva and EPI2ME-16S workflows. Notably, the EPI2ME-16S workflow exhibited the highest Pearson correlation with the microbial standard, minimised misclassification, and increased alignment accuracy. At the species taxonomic level, the BugSeq workflow was superior, with a Pearson correlation coefficient of 0.92.

**Conclusions:**

These findings emphasise the importance of careful selection of PCR settings and a well-structured analytical framework for 16S rRNA full-length gene sequencing. The results showed a robust correlation between the predicted and observed bacterial abundances at both the genus and species taxonomic levels, making these findings applicable across diverse research contexts and with clinical utility for reliable pathogen identification.

**Supplementary Information:**

The online version contains supplementary material available at 10.1186/s12866-024-03208-5.

## Background

Microbiome analysis serves as a cornerstone in deciphering the complexities of ecological systems and human biological functions, encompassing a range of microorganisms, such as bacteria, viruses, archaea, and fungi. The application of phenotypic and genotypic techniques plays a complementary role in understanding bacterial communities. For instance, microscopic examination of bacteria reveals their morphology, size, and spatial arrangement, whereas Gram staining aids in the differentiation of bacterial cells into two classifications based on their cell wall properties, thereby facilitating the identification of specific bacterial species. Although phenotypic data provide valuable information, molecular techniques, such as genotypic analyses, are increasingly being utilised to complement and enhance bacterial characterisation, offering a more comprehensive understanding of microbial communities. Since the 1980s, evidence has shown that non-culturable bacteria often outnumber culturable bacteria, prompting a shift towards DNA sequencing methodologies over traditional culturing approaches for studying microbial communities [1]. Advances in next-generation sequencing (NGS) platforms have had a transformative impact on microbiological analyses [2].

In clinical research, DNA sequencing is increasingly replacing traditional culture-dependent methods, particularly for identifying uncultured bacteria and novel pathogens [3]. This technique offers critical insights into potential correlations between microbiota alterations and various diseases, owing to its ability to reflect the true microbiota composition with high fidelity [4]. Recent studies have linked altered microbiota to various diseases, such as allergies [5] or obesity [6], and neurodegenerative disorders, such as Parkinson’s [7] and Alzheimer’s disease [8]. Moreover, microbiome sequencing is a valuable tool for epidemiological and environmental monitoring. With the rise of globalisation and mounting concerns over emerging diseases, the importance of identifying and tracking the spread of pathogens, as well as monitoring changes in microbial ecosystems over time, can be achieved by DNA sequencing [9]. Furthermore, microbiome sequencing can provide valuable insights into the potential effects of environmental factors on the microbiome, helping to inform policies and promote interventions to protect human health [10] and the environment [11].

A prevalent technique for bacterial classification in microbiome sequencing is amplicon sequencing of the 16S ribosomal RNA (rRNA) gene. This gene encompasses nine variable regions (V1–V9) interspersed with conserved sequences, and serves as a reliable marker for taxonomic identification [12]. Traditional sequencing methods often generate short-read sequences that inadequately cover the full-length 16S rRNA gene, which is approximately 1,500 base pairs (bp) long [13]. Consequently, these methods limit targeted genomic regions, thereby affecting the precision of taxonomic classification. For instance, previous studies have indicated that while the V4-V6 region is more representative of the full-length 16S rRNA gene, the V2 and V8 regions are less reliable [14]. As a result, variable regions are likely to underestimate the true species richness of microbiome samples. Oxford Nanopore Technologies (ONT) MinION sequencer offers the advantage of longer read lengths (up to 2 Mbp), enabling comprehensive analysis of the full-length 16S rRNA gene [15]. Pacific Biosciences, commonly referred to as PacBio, has also demonstrated the capability to sequence long read-length DNA, with an average of over 12 kb. However, acquisition of a PacBio sequencer requires substantial capital investment, which restricts the availability of these technologies to individual laboratories. In contrast, the MinION sequencer is a compact benchtop device that can be directly connected to a laptop and necessitates a relatively modest upfront financial commitment compared to PacBio instruments.

The fundamental concept of ONT is based on the transit of single-stranded DNA molecules through nanopores on a synthetic membrane. As DNA moves through the nanopores, it results in variations in the electrical current across the membrane. These changes correspond to a specific nucleotide base, and the raw electrical signals are converted to digital data, generating a sequence of signals that accurately represents the DNA sequence [16]. Although MinION sequencing is capable of analysing longer reads and examining the entire 16S rRNA gene [17], along with faster processing and examination of the results [18], it also presents challenges, such as lower data yield and increased misclassification rates [19]. Every aspect of the DNA library preparation, from sample collection and storage to DNA isolation, can influence analytical outcomes [20, 21]. Similarly, the choice of bioinformatics pipelines and analysis software has been shown to impact the outcomes. The sequencing procedure is complex, and interpreting the results that reflect the true composition of the microbiota can be a lengthy process.

**Fig. 1:**
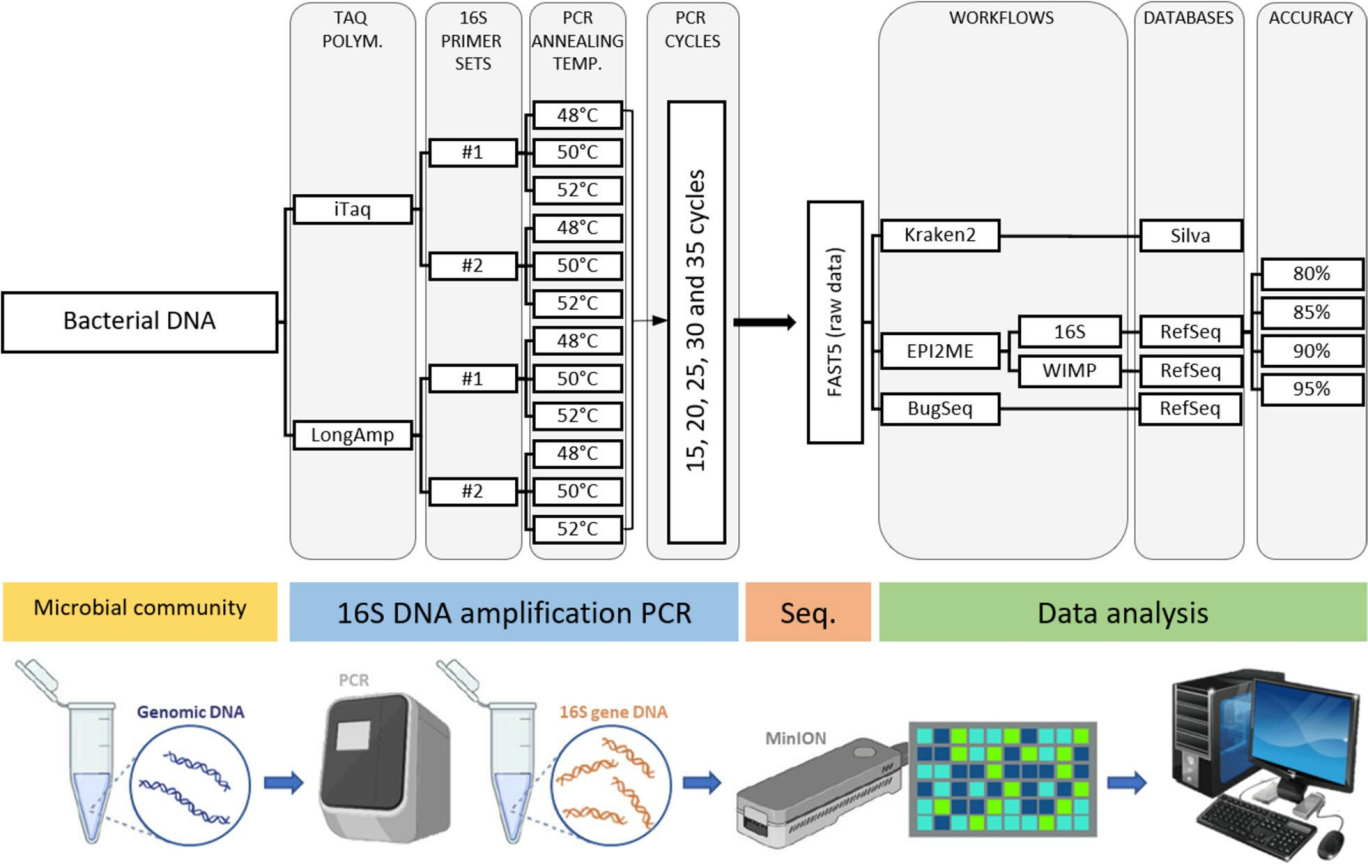
Schematic diagram of the basic principle and process of nanopore sequencing workflow. DNA from a pre-set microbial community is used as a template for 16S rRNA gene amplification by polymerase chain reaction (PCR). We analysed the influence of the type of Taq polymerase, annealing temperature, type of primers, and number of cycles used during the reactions. Following a standard library preparation process, the 16S gene DNA fragment was subjected to sequencing using a nanopore sequencer (MinION), and the results were then screened and processed according to the workflows, databases and accuracy settings. (Diagram adapted from [22])

This study aimed to evaluate the efficacy and reliability of MinION nanopore sequencing for bacterial taxonomic classification, specifically focusing on the full-length 16S rRNA gene. Our objectives included optimisation of 16S rRNA gene sequencing protocols and comparison of bioinformatics workflows and databases for effective bacterial characterisation. We also assessed various methodological factors, such as PCR annealing temperature, primer sequence selection, Taq polymerase PCR cycle numbers, reference databases, and workflows, to optimise the DNA sequencing results (Fig. 1).

## Methods

### DNA amplification and 16S rRNA sequencing

For bacterial samples, we used the ZymoBIOMICS™ Microbial Community Standard, comprising an in vitro mixture of microbial cells containing eight bacterial strains in an already purified bacterial genomic DNA form (Zymo, D6300). The Microbial Community Standard comprised DNA prepared from the following bacterial strains in fixed proportions: *Pseudomonas aeruginosa* (4%; NRRL Acc. No. = B-3509), *Escherichia coli* (10%; NRRL Acc. No. = B-1109), *Salmonella enterica* (10%; NRRL Acc. No. = B-4212), *Lactobacillus fermentum* (18%; NRRL Acc. No. = B-1840), *Enterococcus faecalis* (10%; NRRL Acc. No. = B-537), *Staphylococcus aureus* (16%; NRRL Acc. No. = B-41012), *Listeria monocytogenes* (14%; NRRL Acc. No. = B-33116), and *Bacillus subtilis* (17%; NRRL Acc. No. = B-354). DNA samples were analysed by NGS of 16S ribosomal RNA (rRNA) and barcoded using an adapted protocol by ONT (Protocol PCR barcoding amplicons, SQK-LSK109). Amplicon libraries were generated targeting the hypervariable regions 1–9 (V1–V9) of the 16S rDNA (~ 1,500 bp DNA fragments).

Bacterial DNA was amplified using two sets of 16S universal primers: Set#1, forward primer 27F (5'-AGAGTTTGATCCTGGCTCAG-3') and reverse primer 1492R (5'-CGGTTACCTTGTTACGACTT-3') [23]; Set#2, forward primer GM3 (5'-AGAGTTTGATCMTGGC-3') and reverse primer GM4 (5'-TACCTTGTTACGACTT-3') [24]. Each primer was tagged to enable barcoding using a PCR Barcoding Expansion 1–96 kit (ONT, EXP-PBC096). Following analysis of primer sets using TestPrime 1.0 (https://www.arb-silva.de/search/testprime/) [25], primer Set#2 produced 19 bp longer DNA sequences than Set#1 and allowed for more flexible recognition of bacterial DNA in the locus, with 123,073 matched regions compared to 5,471 matched regions of primer set#1 (Sup. Figure 1). The tags used were 5’-TTTCTGTTGGTGCTGATATTGC-3’ (forward primer) and 5’-ACTTGCCTGTCGCTCTATCTTC-3’ (reverse primer). Two Taq polymerases were used: LongAmp® Hot Start Taq DNA Polymerase (LongAmp DNA Polymerase, New England Biolabs, M0534) and iQ SYBR® Green Supermix (iTaq DNA Polymerase, Bio-Rad, 1708880), both supplied with a ready-to-use master mix. LongAmp is the polymerase recommended by ONT protocols, while iTaq is specifically selected for its rapid PCR amplification rate and ease of use.

**Fig. 2:**
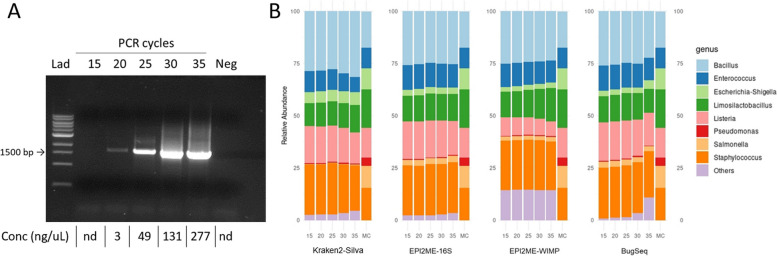
A representative agarose gel (0.8%) showing amplified 16S gene DNA (~ 1,500 bp) after 15-20-25-30-35 PCR cycles. PCR amplification was performed using the LongAmp polymerase and primer set#1. **(A,** DNA concentrations are shown below**)**. In the bar graph, four workflows, namely Kraken2-Silva, EPI2ME-16S, EPI2ME-WIMP, and BugSeq, were compared to determine the relative abundance of microbial genera in the mock community based on increasing PCR cycles **(B)**. (MC = microbial community, others = misclassified sequences)

For the 16S amplification reactions, we combined 2 µL of the primer mix (final concentration of 400 nM), with 1 ng of mock community DNA and 12.5 µL LongAmp or iTaq DNA polymerases for a final volume of 25 µL. Specific products were amplified using a thermal cycler (T3000 Thermocycle, Biometra), with the following settings: 1 min at 94°C for polymerase activation (1 cycle); 20 s at 94°C for denaturation, 30 s at 48°C, 50°C or 52°C for annealing and 90 s at 65°C for extension (15, 20, 25, 30 or 35 amplification cycles); and a final step of 3 min at 65°C. To assess possible contamination, each PCR reaction included a no-template control sample amplified for 35 cycles (this sample did not amplify any DNA fragments, Fig. 2A). Following PCR, the DNA fragments were purified by SPRIselect magnetic beads (Beckman Coulter, B23317) and after purification, the DNA concentration was measured by Qubit dsDNA BR Assay Kit using Qubit 4.0 fluorimeter (Thermo Fisher Scientific, Q33238).

### Barcoding and library preparation

Following 16S amplification, the DNA was barcoded using a specific protocol. Briefly, 1 µL of the barcoding primer mix, 11.5 µL of 16S amplified DNA (for a total amount of 0.5 nM of DNA) and 12.5 µL LongAmp® Hot Start Taq DNA Polymerase were mixed in a final volume of 25 µL. The PCR reaction was carried out using the following settings: 3 min at 94°C for polymerase activation (1 cycle); 15 s at 94°C for denaturation, 15 s at 62°C for annealing and 100 s at 65°C for extension (12 amplification cycles); and a final step of 3 min at 65°C. Barcoded DNA was then purified using SPRIselect magnetic beads and measured by Qubit dsDNA BR Assay Kit using Qubit.

At this stage, the different barcoded DNA were pooled together (final 1 μg of multiple barcoded DNA) and processed for end repair and dA-tailing using the NEBNext® Companion Module for ONT Ligation Sequencing (New England Biolabs, E7180S). After a purification step using SPRIselect magnetic beads, Adapter Bead Binding buffer was added to the DNA library. After quality control and priming of the flow cell (Flow Cell Mk I, R9.4, FLO-MIN106D), the purified DNA library ( 50 fmol) was loaded, and a standard sequencing protocol was initiated using the MinION Mk1C device (ONT, MIN-101C).

### DNA sequence analysis

Basecalling was performed with the Guppy agent (version 6.3.7) integrated into the EPI2ME software (version 5.2.13, ONT), and FAST5 files were converted to FASTQ files. Barcodes were trimmed, and sequences were filtered to include only those with a q-score ≥ 9. The output FASTQ files were uploaded to BugSeq, a commercially available platform workflow (version 1.1, database version: RefSeq September 2022) [26] for 16S sequence classification [27, 28]. The FASTQ files were similarly processed using Kraken2 [29] based on the SILVA database [30] as a reference. Finally, the same files were uploaded to EPI2ME using the "16S" workflow (EPI2ME-16S) and the "what's in my pot" (EPI2ME-WIMP) workflow, and the minimal accuracy level was set to 77%. The sequencing data in the EPI2ME workflows are processed by cloud-based computational infrastructure, incorporating demultiplexing, quality control, and taxonomic assignment using the BLAST algorithm against the Reference Sequence (RefSeq) database [31], which is an open access, annotated, and curated collection of publicly available nucleotide sequences built by the National Center for Biotechnology Information (NCBI). Reads shorter than 1,000 bp and longer than 1,850 bp were discarded.

### Statistical analysis

The data was analysed using R (version 4.2.2). At the genus level, descriptive analyses were performed using proportions. The Pearson correlation coefficient (r) was used to determine the linear correlation, and to measure the strength and direction of the relationship between the analyses and the theoretical composition of the bacterial community. Accuracy represents the percentage of identical matches in an alignment. A heatmap of differential abundance was created using NG-CHM GUI v.2.20.2 BUILDER, using Euclidean distance as the distance measure and Ward's method as a linkage rule [32]. At the species level, multi-layered pie charts were created using Krona [33] on the BugSeq web-based portal.

## Results

### Impact of PCR cycles, workflows and databases choices on bacterial population identification

We investigated the effect of the number of PCR cycles on DNA sequencing accuracy by amplifying the V1-V9 region of the 16S rRNA gene using LongAmp polymerase and MinION sequencing. Our findings indicated a correlation between the number of amplification cycles and the corresponding increase in DNA yield. Starting from undetectable agarose gel electrophoresis amounts after 15 cycles, the quantity increased to 277 ng/µL after 35 cycles (Fig. 2A).

After barcoding, sequencing was performed by using equal amounts of DNA from each sample. Subsequently, the relative abundance of bacteria at the genus level was determined using four different workflows, namely Kraken2-Silva, EPI2ME-16S, EPI2ME-WIMP, and BugSeq.(Fig. 2B). The increasing number of PCR cycles negatively influenced taxa identification in all workflows, increasing the percentage of misclassified reads (either unclassified or incorrectly classified). The EPI2ME-WIMP workflow displayed the highest misclassified proportion, with a constant average of 14% (Table 1).

**Table 1:** Percentage of misclassified reads per workflow and the number of PCR cycles. Misclassified reads included all false positives, unclassified reads, and those classified higher than the genus classification

16S Databases		**Silva**	**RefSeq**		
Workflows		**Kraken2**	**EPI2ME**		**BugSeq**
			**16S**	**WIMP**	
**PCR cycles**	**15**	2.6%	2.3%	14.3%	0.8%
	**20**	2.7%	2.2%	14.6%	1.3%
	**25**	2.8%	2.4%	14.7%	1.5%
	**30**	3.4%	2.7%	14.4%	3.5%
	**35**	4.4%	3.5%	14.3%	10.8%

The Epi2me and 16S workflow had a minimum identification accuracy of 80%. PCR was conducted using an annealing temperature of 48°C, primer set#1, and LongAmp Taq polymerase

In contrast, the BugSeq workflow yielded the most accurate results when utilising a range of 15–25 PCR cycles, with an average misclassification rate of 1.2%. Consistently, *Pseudomonas* and *Lactobacillus* were the organisms that were least identified among all workflows, while *Bacillus* was over-identified. We do not attribute the disparities in identification to the proportion of each bacterial species in the mock community, as *Bacillus* and *Lactobacillus* have similar percentages (17% and 18%, respectively), contrary to *Pseudomonas* (4%). *Salmonella* is under-represented by Kraken2-Silva. The total number of genera identified was highest using EPI2ME-WIMP (over 250 genera) and lowest using BugSeq (8 genera, Table 2).

**Table 2:** Number of genera identified per workflow and number of PCR cycles. No minimal thresholds were set

16S Databases		**Silva**	**RefSeq**		
Workflows		**Kraken2**	**EPI2ME**		**BugSeq**
			**16S**	**WIMP**	
**PCR cycles**	**15**	71	197	252	8
	**20**	74	206	265	8
	**25**	89	211	291	8
	**30**	76	214	286	8
	**35**	84	209	261	9

The Epi2me and 16S workflow had a minimum identification accuracy of 80%. PCR was conducted using an annealing temperature of 48°C, primer set#1, and LongAmp Taq polymerase

The Kraken2-Silva, EPI2ME-16S, and BugSeq workflows had comparable Pearson correlation values (Table 3). Kraken2-Silva (mean *r* = 0.73) and EPI2ME-16S (mean *r* = 0.77) consistently maintained a stable correlation as PCR cycles increased, while the BugSeq correlation decreased at 35 PCR cycles (*r* = 0.58). EPI2ME-WIMP workflow consistently exhibited the weakest correlation across all PCR cycles (mean *r* = 0.46).

**Table 3:** Correlation between the expected and observed proportions of relative abundance at the genus level and the number of PCR cycles. Correlations were calculated using the Pearson’s correlation coefficient

16S Databases		**Silva**	**RefSeq**			
Workflows		**Kraken2**	**EPI2ME**			**BugSeq**
			**16S**		**WIMP**	
**PCR cycles**	**15**	0.73	0.77	0.46		0.79
	**20**	0.74	0.77	0.45		0.78
	**25**	0.74	0.78	0.45		0.79
	**30**	0.74	0.77	0.47		0.75
	**35**	0.73	0.76	0.49		0.58

The Epi2me and 16S workflow had a minimum identification accuracy of 80%. PCR was conducted using an annealing temperature of 48°C, primer set#1, and LongAmp Taq polymerase

The EPI2ME-16S workflow can be further improved by increasing the alignment accuracy (Sup. Figure 2). The highest correlation, with minimal misclassification, was achieved by including only sequences with an accuracy greater than 95% (0.1% misclassified or unclassified, and Pearson correlation *r* = 0.82); however, this setting accounted for only ~ 25% of all reads that aligned to the reference database.

### Effect of primer pair set and Taq Polymerase on 16S gene DNA amplification

We compared two sets of primers, Set#1 and Set#2, for amplification of the 16S rRNA gene, along with two Taq polymerases. Primer Set#1 produced equally longer DNA sequences than Set#2, however the type of Taq polymerases used influenced the length, 1,457 bp with LongAmp and 1,449 bp with iTaq (Table 4). Compared to iTaq polymerase (2.2 × 10^4^ DNA sequences), LongAmp was producing the larger number of sequences with both primer sets, Set#1 (2.5 × 10^4^ DNA sequences) and Set#2 (3.4 × 10^4^ DNA sequences).

**Table 4:** Comparison of different PCR annealing temperatures, primer sets, and Taq polymerases, and their influence on DNA amplification

		**Polymerases**			
		**LongAmp**		**iTaq**	
**Primer set**	**Annealing Temperature** **(°C)**	**Read length** **(bp)**	**Sequence** **Number (× 10**^**4**^**)**	**Read length** **(bp)**	**Sequence** **Number (× 10**^**4**^**)**
Set#1	48	1′457	2.35	1′451	2.10
	50	1′458	2.99	1′450	2.69
	52	1′455	2.20	1′447	2.05
**Average ± SD**		**1′457 ± 1.5**	**2.51 ± 0.42**	**1′449 ± 2.1**	**2.28 ± 0.36**
Set#2	48	1′458	2.92	1′451	2.15
	50	1′454	3.68	1′448	2.86
	52	1′455	3.45	1′448	1.40
**Average ± SD**		**1′456 ± 2.1**	**3.35 ± 0.39**	**1′449 ± 1.7**	**2.14 ± 0.73**

Equal amounts of DNA were sequenced for each sample

### Comparison of different PCR annealing temperatures and Taq polymerases on sequencing accuracy

**Fig. 3:**
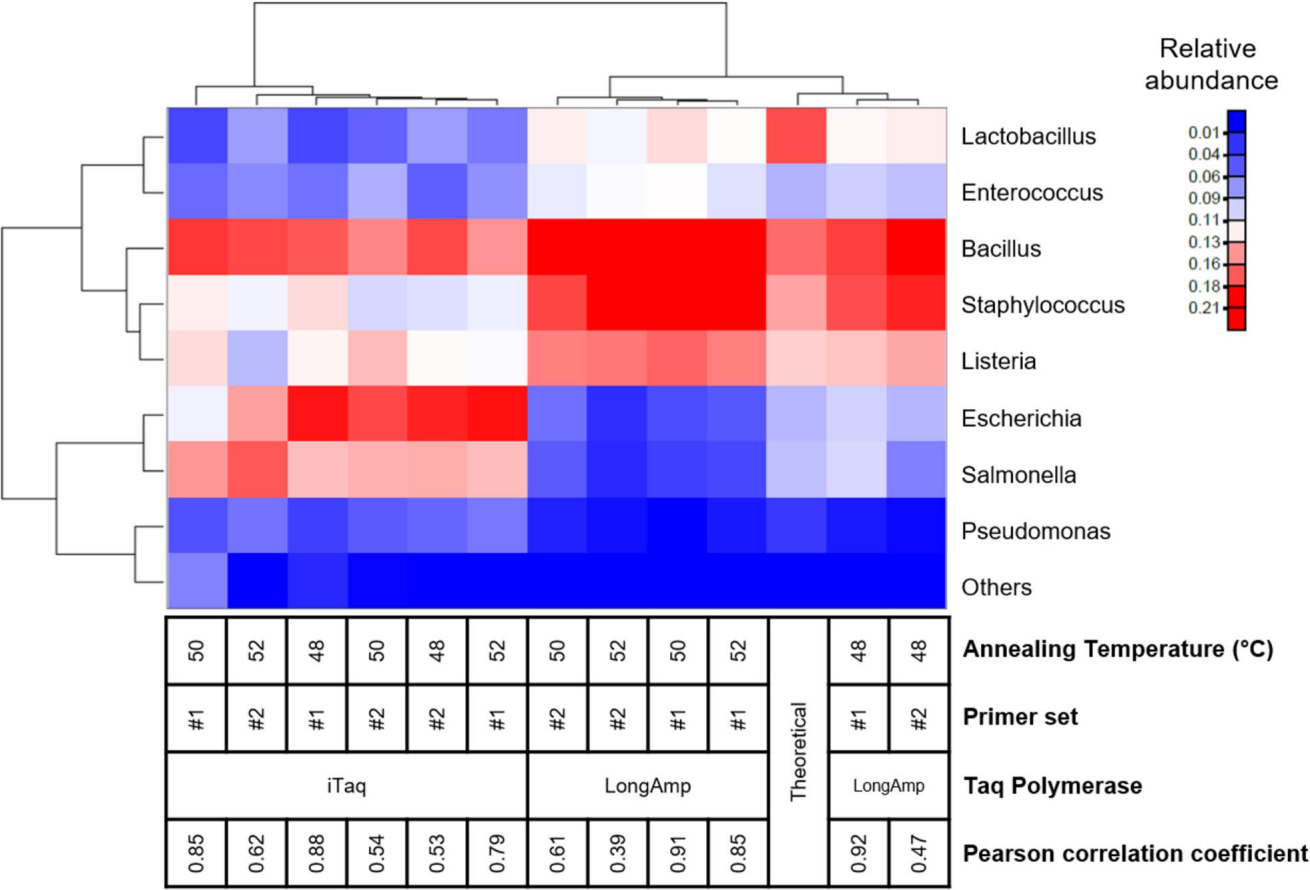
The hierarchical clustering heat map shows the relative abundance of each genus in the microbial mock community using primer sets #1 and #2 at different annealing temperatures (48, 50, and 52°C) and Taq polymerases (LongAmp and iTaq). The results were analysed using BugSeq, and the bottom row of the table shows the Pearson correlation coefficient. The results were compared with the expected (theoretical) proportion of the mock community. The coloured gradient legend represents a linear scale of relative abundance. (others = misclassified sequences)

We compared the influence of two Taq polymerases, LongAmp and iTaq, on sequencing accuracy at various PCR annealing temperatures (48°C, 50°C, and 52°C). The results were analysed using BugSeq, and the most accurate results were obtained when the annealing temperature was set at 48°C (Fig. 3). Increasing the PCR annealing temperature reduced the accuracy of LongAmp Taq polymerase, while the iTaq results remained unaffected. iTaq polymerase effectively amplified gram-negative bacteria such as *Pseudomonas sp.*, *Escherichia sp.*, and *Salmonella sp.;* however*,* it showed a greater proportion of unclassified or misclassified bacteria when compared to the results obtained using LongAmp Taq polymerase. The highest correlation with the pre-set proportions of the mock community was obtained at an annealing temperature of 48°C, primer set#1 and LongAmp Taq polymerase (*r* = 0.92). With iTaq polymerase, the correlation coefficient was higher when primer set#1 was used (approximately 50% more accurate).

### Effect of primer pair set and taq polymerase on microbiome composition detection at the species level

**Fig. 4:**
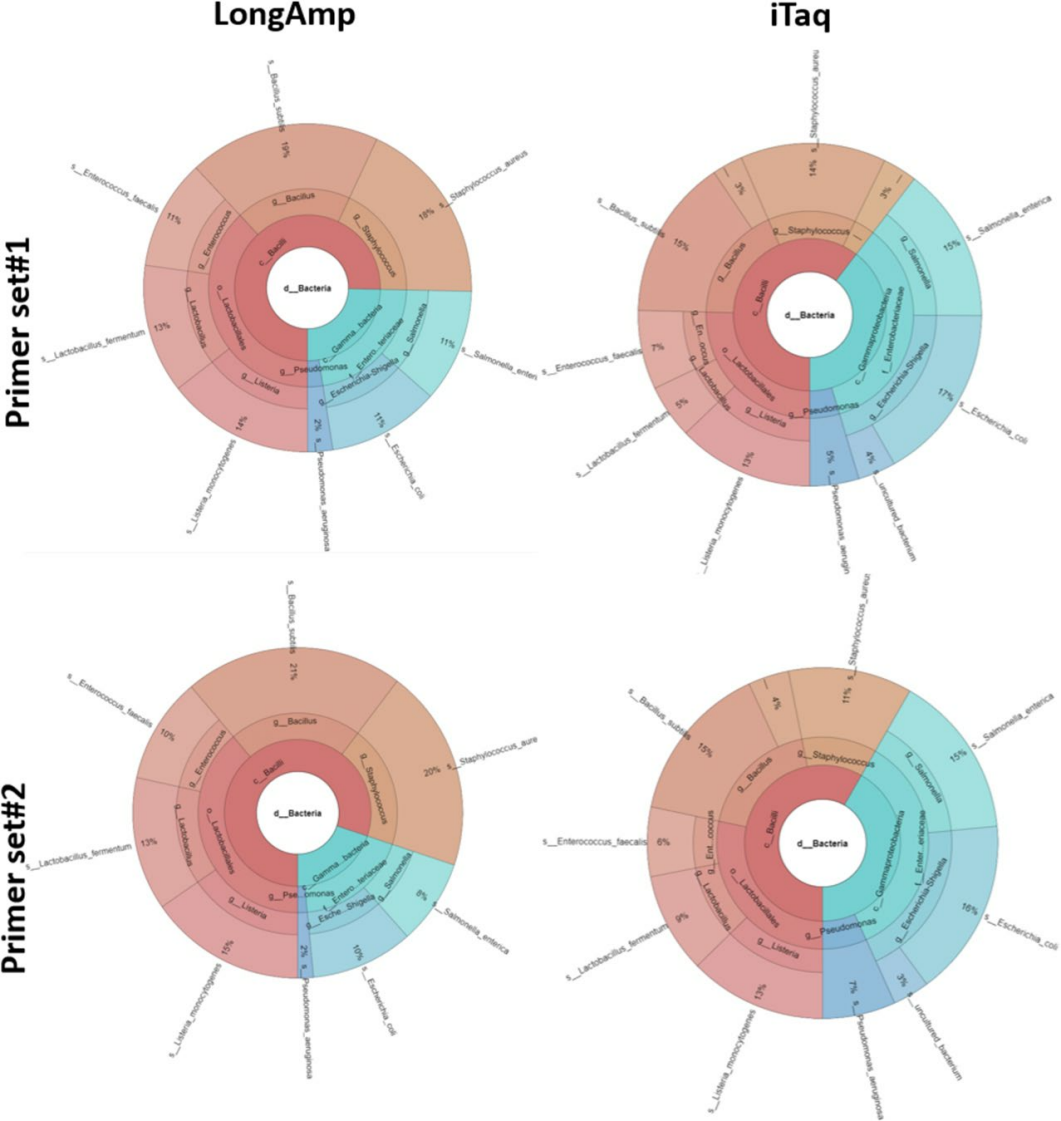
Comparative analysis was performed to determine the most effective combination for accurate species identification of the two primer sets, Set#1 and Set#2, and the two Taq Polymerases, LongAmp and iTaq. The analysis was conducted using BugSeq at an annealing temperature of 48°C. The relative abundance of each species is presented as %

We compared two sets of primers, Set#1 and Set#2, and two Taq polymerases, LongAmp and iTaq, for the amplification of the 16S rRNA gene for annotation accuracy at the species level. The PCR was conducted with an annealing temperature of 48°C and the analysis with the BugSeq workflow, and it showed that the primer set used had no impact when employing the LongAmp polymerase, as both primer sets demonstrated a Pearson correlation coefficient of *r* = 0.91 (Fig. 4). However, primer set#2 was more accurate than primer set#1 when used with iTaq polymerase (*r* = 0.61 compared to *r* = 0.47). None of the other workflows could be used at the species level owing to insufficient accuracy and reliability of the results (data not shown).

## Discussion

In this study, we introduced a streamlined methodology to facilitate the sequencing of bacterial composition in complex DNA samples that can be seamlessly integrated into existing laboratory workflows and various research fields, including clinical settings. The utilisation of ONT nanopore sequencing has provided the opportunity to conduct real-time sequencing and data analysis at a cost-effective rate [34] to diagnose bacterial infections [35, 36]. To effectively implement the MinION sequencer as a rapid diagnostic tool, it is crucial that future investigations adhere to well-established methodologies that address a range of possible biases. We noticed that the choice of workflow and database for reference had a substantial influence on the results, as corroborated by the existing literature. For example, in the case of 16S rRNA gene amplification by PCR, the accuracy of identifying bacteria in a community can vary depending on multiple factors, such as the Taq polymerase and primers used, the number of cycles used during amplification, and the workflows and databases used for classifying the DNA sequences. Bacterial DNA, already purified, was obtained from a pre-existing mock community to minimise potential sources of bias, including variations in sampling and storage procedures [37], and differences in DNA extraction methods [38].

The selection of a workflow, including the methodology for classifying the cleaned and filtered sequences and the reference database, has a significant impact on the outcomes, as evidenced by previous publications [39]. In our study, BugSeq, EPI2ME-16S, and Kraken2-Silva workflows performed well in classifying the sequences at the genus level with an observed error rate and proportion of unclassified reads below 5% and demonstrated a comparable Pearson correlation. In particular, the BugSeq workflow employs a stringent identification approach to determine bacterial taxa, requiring a minimum of 200 reads for taxonomic classification. Additionally, this method demonstrated high precision in accurately identifying bacterial species, in contrast to other methods, and SILVA can only annotate at the genus level. The EPI2ME-16S and Kraken2-Silva workflows have been shown to effectively classify bacteria at the genus level, and the EPI2ME-16S workflow outcomes can be improved by changing the alignment accuracy in the settings. For example, we found that the percentage of misclassified reads was < 1% when a minimum accuracy threshold of 95% was applied. However, the proportion of sequenced reads included in the analysis was less than 50% of the total. The presence of misclassified reads has a significant impact on the reporting of diversity indices, particularly in the case of indices that rely on singleton groups and place an emphasis on rare taxa. As such, minimising misclassifications leads to improved accuracy of the reported diversity index.

One intriguing aspect that merits discussion is the effect of varying the number of PCR cycles on the accuracy of bacterial identification. It was observed that an increased number of PCR cycles, beyond 25, led to compromised identification accuracy; specifically, when using a starting DNA template of 1 ng in a 25 μL reaction mixture, the identification process became increasingly imprecise. This is in agreement with previous studies that observed a similar trend due to sequence saturation [23] and generation of chimeric amplification products (a single DNA amplicon consisting of sequences derived from various 16S rRNA genes rather than just one) [40]. Therefore, to ensure the accurate analysis of bacterial populations, we recommend employing effective reaction conditions to minimise the number of required PCR amplification cycles. For example, in the case of the gut microbiome, which is characterised by significant bacterial diversity, a low number of PCR cycles will provide a more rigorous, precise, and robust analysis of the data. However, when examining the cutaneous microbiome, where bacterial abundance is notably lower, an increase in PCR cycles may serve as a critical factor, as a higher number of amplification cycles is needed to detect all bacterial species. However, a greater number of amplification cycles increases the likelihood of false positives.

Furthermore, the choice of a primer set for 16S rRNA sequencing is essential for creating DNA libraries and analysing bacterial composition [41]. Primer bias resulting from differential annealing can result in the over- or under-representation of specific taxa. Furthermore, certain bacteria may not be detected if their consensus sequences do not align well with the primer set used [42]. In our protocol, Set#1 (27F-1492R) binds DNA in the proximity of Set#2 (GM3-GM4), with the latter characterised by a shorter base pair sequence to provide primers with more flexibility to attach to bacterial DNA, thereby expanding the diversity of the sampled population. However, our analysis showed that not only did the amplified DNA sequences had the same length (1,456 bp), but Set#2 demonstrated inferior precision in the determination of bacterial identity compared to Set#1. These results show that even minor modifications to the primer set can lead to significant consequences. Therefore, it is crucial to be cautious when comparing relative abundance across various studies.

Furthermore, we observed that the type of polymerase enzyme used in PCR significantly affected the bacterial community profile. Specifically, iTaq polymerase was found to favour 16S gene amplification in gram-negative bacteria, altering the analysis of the bacterial composition within the population. Both polymerases, iTaq and LongAmp, required a hot-start initiation step to enhance the specificity and sensitivity of the reaction. iTaq provided excellent yield when amplifying products as small as 200–300 bp up to > 2Kbp, and genomic DNA can be amplified up to 5 kb. The LongAmp polymerase can generate up to 30 kb, with high specificity and throughput. The difference between the two polymerases is in the 3´ → 5´ exonuclease activity of the Deep Vent DNA polymerase present in the LongAmp amplification mix, which increases the fidelity and robust amplification of Taq Polymerase [43]. However, the different concentrations of magnesium chloride (MgCl_2_) included in the PCR reactions might have affected the selectivity of DNA amplification. A DNA sequence enriched with guanine-cytosine (GC) content, such as that in gram-negative bacteria (average 55%) compared to gram-positive bacteria (average 41%), often requires a higher magnesium concentration to facilitate primer annealing and DNA polymerase activity. The higher concentration of MgCl_2_ in the iTaq reaction mix (3 mM) than in LongAmp (2 mM) might have favoured primer-template binding and improved DNA polymerase activity in gram-negative bacteria. The increased magnesium ion concentration promoted the denaturation of GC-rich DNA segments and facilitated their amplification. This distinction emphasises the importance of selecting a suitable buffer formulation to achieve desired amplification outcomes in PCR applications.

Selection of the most appropriate protocol also depends on the variability of a particular sample. Finding optimal criteria for cut-off values and setting thresholds present a challenge because of the complexity of understanding the biological meaning of the results. The adoption of predefined thresholds, such as a relative abundance cut-off of 0.01%, is widely used in microbiome research. Despite its wide application, the underlying reasoning for selecting these thresholds remains largely subjective and requires further investigation, particularly regarding the potential influence of false-positive data. To avoid the recurrence of false positive results, we recommend the use of negative and positive controls, implementation of spiking for accurate quantification of bacterial abundance, and use of statistical software to identify potential contaminations [44].

The implementation of these controls may enhance the precision of bacterial community identification and must be associated with the factors analysed and identified in this study. However, it is crucial to consider the limitations of this study, such as the use of a mock community. The mock community includes Gram-positive and Gram-negative bacteria, which introduces similar to the real case variability of studied genomes, but does not guarantee that all bacteria would be accurately identified in diverse environmental conditions, such as faecal samples, urine, or skin swabs. Furthermore, the choice of DNA library preparation protocol should be tailored to the sequencing platform and specific sample types under investigation. For example, EPI2ME workflows are limited by the capacity to customise workflow parameters, including reference databases and alignment preferences, and workflows can be used only by ONT customers through a web-based application. Additionally, the selection of a suitable database for bacterial identification is a crucial factor that can greatly influence the accuracy and efficiency of the process, and this choice is often dependent on available resources. For example, database comparisons showed that SILVA and RefSeq outperformed Greengenes in terms of accuracy (databases not included in this study) [45].

## Conclusions

The present study demonstrates the analytical advantages of employing the MinION nanopore technology for 16S rRNA gene sequencing, notably in achieving a high level of discrimination among closely related bacterial taxa. By optimising various elements in the sequencing process, including PCR cycle numbers, primer sets, Taq polymerases, and bioinformatic workflows, our study contributes to the generation of more robust and reliable data on microbial community compositions (Table 5). By refining the methodology, these optimisations lead to more reliable results and better representation of the microbial community composition in the analysed samples. For genus-level identification, PCR amplification with LongAmp polymerase and primer Set#1 (27F-1492R) at an annealing temperature of 48°C, followed by EPI2ME-16S analysis, yielded accurate results (Appendix S1). The BugSeq workflow was the most efficient for species-level taxonomic assignment.

**Table 5:** The side-by-side analysis of the sequencing variables considered in this study provides a comprehensive evaluation of their respective strengths and limitations

Variables	Advantages	Disadvantages
PCR cycles		
Low number	• Reduced PCR amplification time • More accurate reads	• Loss of identification of rare species in the sample
High number	• Allows detection of bacteria in low-biomass samples, such as skin or urine	• Requires longer time • Introduction of misclassified reads
Annealing Temperature		
48°C	• Sequencing results more accurate than 52°C temperature with LongAmp polymerase	
52°C	• Minimal differences in accuracy with iTaq polymerase	• Reduced amplification of Gram-negative bacteria with LongAmp polymerase
Primer sets		
#1 (27F-1492R)	• Universally recognized primers for 16S PCR amplification • Minimal differences in accuracy with set#2 when used with LongAmp polymerase	• With iTaq polymerase, sequencing accuracy lower than set#2
#2 (GM3-GM4)	• With iTaq polymerase, sequencing accuracy higher than set#1 • With LongAmp polymerase, minimal differences in accuracy compared to set#1	
Taq Polymerases		
LongAmp	• Higher processivity than iTaq • Sequencing results more accurate • Recommended by ONT protocols	
iTaq	• Results are not affected by the annealing temperature (range 48–52°C) • Lower price	• Substantially favour amplification of Gram-negative bacteria • Greater proportion of misclassified bacteria compared to LongAmp polymerase
Workflows		
BugSeq	• Allows for accurate bacterial identification at both genus and species levels	• Subject to payment • Increasing PCR cycles significantly enhanced the percentage of misclassified read
EPI2ME-16S	• Allows for accurate bacterial identification at genus level	• Limited capacity of customization • Workflow can be used only by ONT customers
EPI2ME-WIMP		• Unsuitable for 16S based bacterial identification • Highest percentage of misclassified reads • Limited capacity of customization • Workflow can be used only by ONT customers
Kraken2	• Allows for accurate bacterial identification at genus levels • Free of use workflow	• *Salmonella* is under-represented
16S Databases		
RefSeq	• Non-redundant database • NCBI-managed database compiled from GenBank sequences	
Silva	• Small and large rRNA subunits database including 16S rRNA sequences from the European Nucleotide Archive	
Accuracy		
Low (> 80%)	• Analysis of all reads, including rare bacterial species	• Inclusion in the analysis of possible misclassified reads (~ 3% of the total)
High (> 95%)	• Reliable sequencing results, no misclassified reads	• Over 50% loss of reads • Loss of depth in the analysis

*NCBI* National Centre for Biotechnology Information

Our study serves as a resource for optimising the experimental protocols in microbial genomics and clinical microbiology. This underscores the nuanced impacts of methodological choices on the results and highlights the need for careful experimental design and execution. Thus, optimising 16S rRNA gene sequencing protocols will pave the way for more precise microbial research and diagnostics, facilitating timely patient management and therapeutic interventions.

### Supplementary Information

**Additional file 1:** **Figure S1.** Analysis of primer pairs using TestPrime 1.0. **Figure S2.** Influence of accuracy setting within the Epi2me 16S workflow on the analysis and different PCR cycles. The analysis included the percentage of misclassified reads **(A)**, the correlation to the pre-set microbial community of the relative abundance **(B)**, and the percentage of sequences included in the analysis **(C)**. **Appendix S1**. Optimized Protocol for 16S rRNA Gene Amplification and Sequencing with MinION Nanopore Technology.

## Data Availability

The datasets generated and analysed in the current study are available at the NCBI Sequence Read Archive (SRA) repository, BioProject ID: PRJNA1036127.
