# Supplementary material for: Optimized bacterial community characterization through full-length 16S rRNA gene sequencing utilizing MinION nanopore technology
Source: BMC Microbiol. 2024 Feb 16;24:58. doi: 10.1186/s12866-024-03208-5 (PMC10870487; doi:10.1186/s12866-024-03208-5)
Supplement: Supplementary file 1 — Additional file 1: Figure S1. Analysis of primer pairs using TestPrime 1.0. Figure S2. Influence of accuracy setting within the Epi2me 16S workflow on the analysis and different PCR cycles. The analysis included the percentage of misclassified reads (A), the correlation to the pre-set microbial community of the relative abundance (B), and the percentage of sequences included in the analysis (C). Appendix S1. Optimized Protocol for 16S rRNA Gene Amplification and Sequencing with MinION Nanopore Technology. [file 12866_2024_3208_MOESM1_ESM.docx]

**Optimized Bacterial Community Characterization through Full-length 16S rRNA Gene sequencing utilizing MinION Nanopore Technology**

By Alessandro Bertolo, Ezra Valido and Jivko Stoyanov

**Figure S1**. Analysis of primer pairs using TestPrime 1.0 (<https://www.arb-silva.de/search/testprime/>)


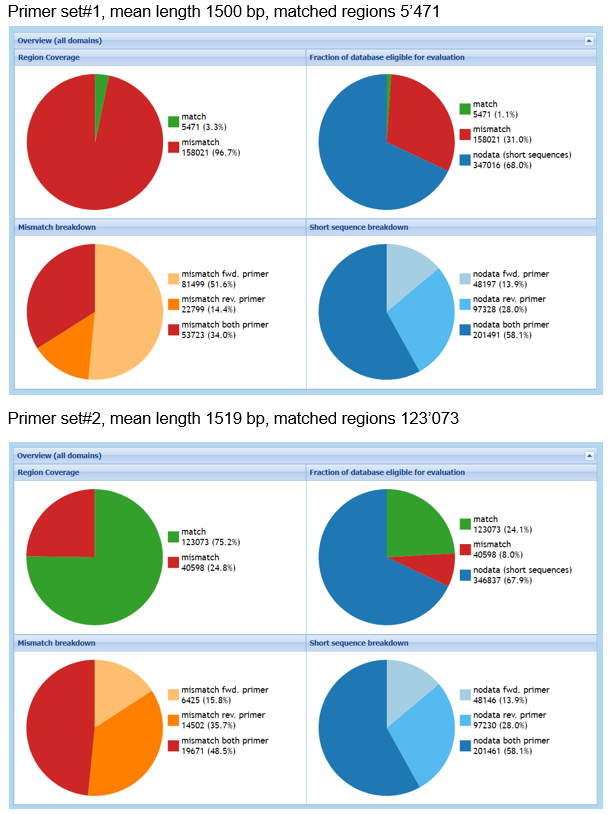


**Figure S2.** Influence of accuracy setting within the Epi2me 16S workflow on the analysis and different PCR cycles. The analysis included the percentage of misclassified reads **(A)**, the correlation to the pre-set microbial community of the relative abundance **(B)**, and the percentage of sequences included in the analysis **(C)**.


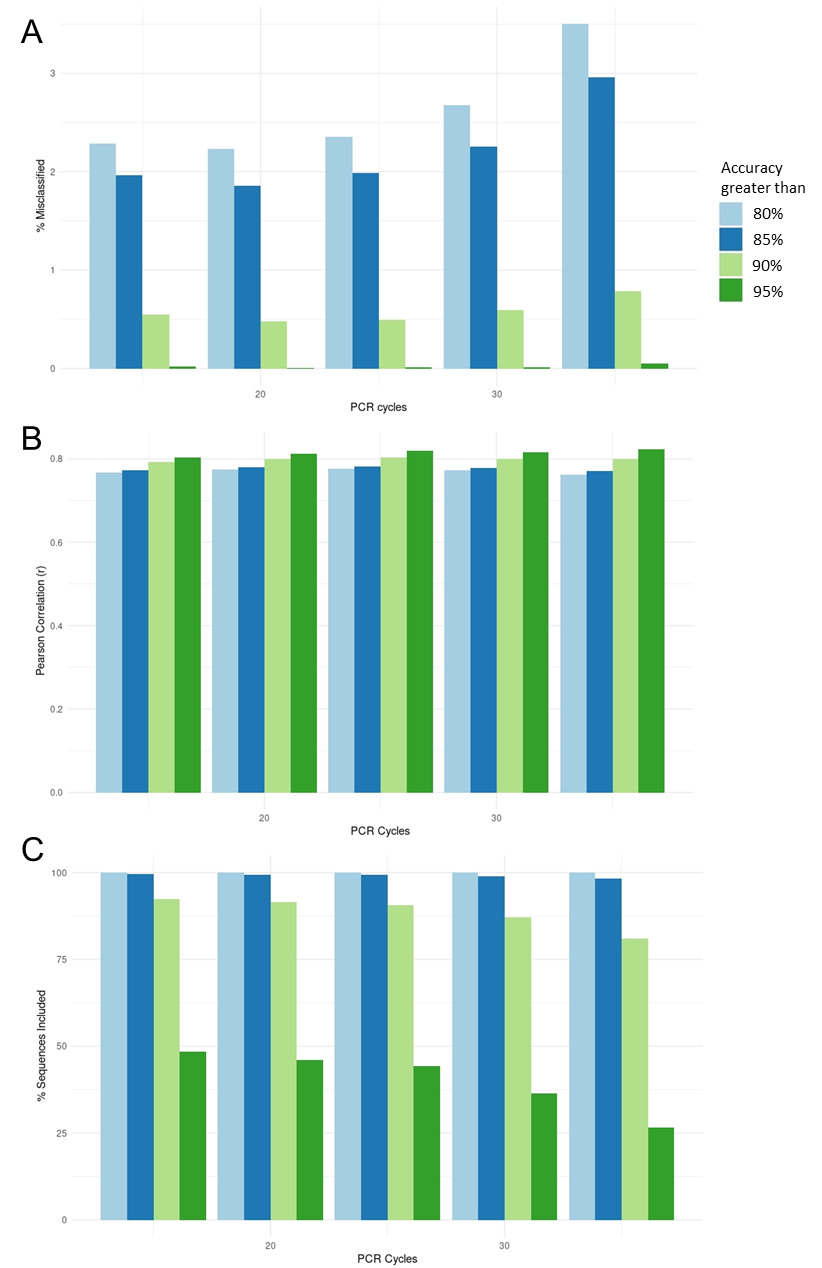


**Appendix S1**. Optimized Protocol for 16S rRNA Gene Amplification and Sequencing with MinION Nanopore Technology.

Adapted from ONT protocol: PCR barcoding (96) amplicons (SQK-LSK109).

**Materials:**

- MinION Mk1C device (ONT, MIN-101C)
- Flow Cell Priming Kit (ONT, EXP-FLP002)
- PCR Barcoding Expansion Pack 1-96 (ONT, EXPPBC096)
- Ligation Sequencing Kit (ONT, SQK-LSK109)

**Consumables:**

- LongAmp® Hot Start Taq DNA Polymerase (New England Biolabs, M0534)
- SPRIselect magnetic beads (Beckman Coulter, B23317)
- Qubit dsDNA BR Assay Kit (Thermo Fisher Scientific, Q32850)
- NEBNext® Companion Module for ONT Ligation Sequencing (New England Biolabs, E7180S)
- Flow cell (Flow Cell Mk I, R9.4, ONT, FLO-MIN106D)
- 1.5 mL Eppendorf DNA LoBind tubes
- 0.2 mL thin-walled PCR tubes
- Nuclease-free water
- Freshly prepared 70% ethanol in nuclease free water

**Equipment:**

- Qubit 4.0 fluorimeter (Thermo Fisher Scientific, Q33238).
- Timer
- Thermal cycler
- Microfuge
- Pipettes and pipette tips P2, P10, P20, P100, P200, P1000

**Protocol**

*16S rRNA gene amplification*

- In a 0.2 ml thin-walled PCR tube, mix the following:
  - 9.5 μL Nuclease-free water
  - 1 μL Bacterial DNA (1 ng)
  - 2 μL primer set#1 (final concentration of 400 nM)
  - 12.5 μL LongAmp Taq HS 2X master mix
- Amplify using the following cycling conditions:
  - Initial denaturation 1 min @ 94 °C (1 cycle)
  - Denaturation 20 secs @ 94 °C (25 cycles)
  - Annealing 30 secs @ 48 °C (25 cycles)
  - Extension 90 secs @ 65 °C (25 cycles)
  - Final extension 3 mins @ 65 °C (1 cycle)
- Transfer the sample to a 1.5 ml DNA LoBind Eppendorf tube and purify the DNA with SPRIselect magnetic beads.
- Resuspend DNA in 30 μL of nuclease-free water.
- Quantify 1 μL of eluted sample using a Qubit fluorimeter.

*Barcoding PCR*

- In a 0.2 ml PCR tubes, set up a barcoding PCR reaction as follows for each library:
  - 1 μL PCR Barcode (EXPPBC096)
  - 11.5 μL 0.5 nM 16S rRNA PCR product
    - ~1'500 bp DNA fragment is equivalent to 12 ng PCR product
  - 12.5 μL LongAmp Taq HS 2x master mix
- Amplify using the following cycling conditions:
  - Initial denaturation 3 mins @ 94 °C (1 cycle)
  - Denaturation 15 secs @ 94 °C (12 cycles)
  - Annealing 15 secs @ 62 °C (12 cycles)
  - Extension 100 secs @ 65 °C (12 cycles)
  - Final extension 3 mins @ 65 °C (1 cycle)
- Purify the barcoded DNA using SPRIselect magnetic beads.
- Quantify the barcoded library using a Qubit fluorometer, and pool all barcoded libraries in the desired ratios in a 1.5 ml DNA LoBind Eppendorf tube.
  - total 1 μg of pooled barcoded libraries in 48 μL Nuclease-free water.

*DNA repair and end-prep*

- Prepare the NEBNext FFPE DNA Repair Mix and NEBNext Ultra II End repair / dA-tailing Module reagents in a 0.2 ml thin-walled PCR tube, and mix the following:
  - 48 μL DNA
  - 3.5 μL NEBNext FFPE DNA Repair Buffer
  - 2 μL NEBNext FFPE DNA Repair Mix
  - 3.5 μL Ultra II End-prep reaction buffer
  - 3 μL Ultra II End-prep enzyme mix
- Using a thermal cycler, incubate at 20°C for 5 min, and 65°C for 5 min.
- Purify DNA with SPRIselect magnetic beads and resuspend in 61 μL Nuclease-free water

*Adapter ligation and clean-up*

- In a 1.5 ml Eppendorf DNA LoBind tube, mix in the following order:
  - 60 μL DNA sample from the previous step
  - 25 μL Ligation Buffer (LNB)
  - 10 μL NEBNext Quick T4 DNA Ligase
  - 5 μL Adapter Mix (AMX)
- Incubate the reaction for 10 min at RT.
- Purify DNA with SPRIselect magnetic beads and resuspend in 15 μL in Elution buffer (EB).
  - During DNA purification wash the beads with Short Fragment Buffer (SFB).
- Quantify 1 μL of eluted sample using a Qubit fluorimeter.

*DNA sequencing*

- Prepare the flow cell priming mix, and load it into the flow cell avoiding the introduction of air bubbles. Wait for 5 minutes. During this time, prepare the library following the steps below.
- In a new tube, prepare the library for loading as follows:
  - 37.5 μL Sequencing Buffer (SQB)
  - 25.5 μL Loading Beads (LB), mixed immediately before use
  - 12 μL DNA library (containing 50 fmol DNA, diluted in EB)
- Mix the prepared library gently by pipetting up and down immediately prior to loading.
- Add 75 μL of sample to the flow cell in a dropwise fashion and start DNA sequencing on the MinION Mk1C device

*Data analysis*

- Convert FAST5 files to FASTQ files with the Guppy agent integrated into EPI2ME software.
  - The process involves barcode trimming and sequence filtering to ensure only those with a q-score of at least 9 are selected.

For genus analysis:

- Upload the FASTQ files to EPI2ME using the "16S" workflow (EPI2ME-16S), with minimal accuracy level was set to 85%.
  - The process includes demultiplexing, quality control, and taxonomic assignment using the BLAST algorithm against the Reference Sequence (RefSeq) database.
  - Reads shorter than 1'000 bp and longer than 1'850 bp are discarded.

For species analysis:

- The output FASTQ files were uploaded to BugSeq, a commercially available platform workflow
  - The process includes demultiplexing, quality control, and taxonomic assignment against the Reference Sequence (RefSeq) database.
  - Reads shorter than 1'000 bp and longer than 1'850 bp are discarded.
